# Association Between TAS2R38 Gene Polymorphisms and Colorectal Cancer Risk: A Case-Control Study in Two Independent Populations of Caucasian Origin

**DOI:** 10.1371/journal.pone.0020464

**Published:** 2011-06-02

**Authors:** Maura Carrai, Verena Steinke, Pavel Vodicka, Barbara Pardini, Nils Rahner, Elke Holinski-Feder, Monika Morak, Hans K. Schackert, Heike Görgens, Susanne Stemmler, Beate Betz, Matthias Kloor, Christoph Engel, Reinhard Büttner, Alessio Naccarati, Ludmila Vodickova, Jan Novotny, Angelika Stein, Kari Hemminki, Peter Propping, Asta Försti, Federico Canzian, Roberto Barale, Daniele Campa

**Affiliations:** 1 Department of Biology, University of Pisa, Pisa, Italy; 2 Institute of Human Genetics, University of Bonn, Bonn, Germany; 3 Institute of Experimental Medicine, Academy of Sciences of the Czech Republic, Prague, Czech Republic; 4 Institute of Biology and Medical Genetics, First Faculty of Medicine, Charles University in Prague, Czech Republic; 5 Department of Internal Medicine, University Hospital of Ludwig-Maximilians-University, Munich, Germany; 6 Medical Genetics Center/Medizinisch Genetisches Zentrum (MGZ), Munich, Germany; 7 Department of Surgical Research, Technische Universität Dresden, Dresden,Germany; 8 Humangenetik Ruhr-Universität Bochum, Bochum, Germany; 9 Institute of Human Genetics, University of Düsseldorf, Düsseldorf, Germany; 10 Department of Applied Tumour Biology, Institute of Pathology, University of Heidelberg, Heidelberg, Germany; 11 Institute of Medical Informatics, Statistics and Epidemiology, University of Leipzig, Leipzig, Germany; 12 Institute of Pathology, University of Bonn, Bonn, Germany; 13 Department of Oncology, First Faculty of Medicine, Charles University, Prague, Czech Republic; 14 The German Cancer Research Center/Deutsches Krebsforschungszentrum (DKFZ), Heidelberg, Germany; 15 Center for Primary Health Care Research, Clinical Research Center, Lund University, Malmö, Sweden; Brigham & Women's Hospital, and Harvard Medical School, United States of America

## Abstract

Molecular sensing in the lingual mucosa and in the gastro-intestinal tract play a role in the detection of ingested harmful drugs and toxins. Therefore, genetic polymorphisms affecting the capability of initiating these responses may be critical for the subsequent efficiency of avoiding and/or eliminating possible threats to the organism. By using a tagging approach in the region of Taste Receptor 2R38 (*TAS2R38*) gene, we investigated all the common genetic variation of this gene region in relation to colorectal cancer risk with a case-control study in a German population (709 controls and 602 cases) and in a Czech population (623 controls and 601 cases). We found that there were no significant associations between individual SNPs of the *TAS2R38* gene and colorectal cancer in the Czech or in the German population, nor in the joint analysis. However, when we analyzed the diplotypes and the phenotypes we found that the non-taster group had an increased risk of colorectal cancer in comparison to the taster group. This association was borderline significant in the Czech population, (OR = 1.28, 95% CI 0.99–1.67; P_value_ = 0.058) and statistically significant in the German population (OR = 1.36, 95% CI 1.06–1.75; P_value_ = 0.016) and in the joint analysis (OR = 1.34, 95% CI 1.12–1.61; P_value_ = 0.001). In conclusion, we found a suggestive association between the human bitter tasting phenotype and the risk of CRC in two different populations of Caucasian origin.

## Introduction

Colorectal cancer (CRC) is the third most common cancer in the world and the second in Europe [1], [2]. Genetic background is thought to play a role in modulating individual risk [3]. The main interest of the research on the genetic susceptibility of CRC has been focused on genes involved in xenobiotic transport and metabolism [4], [5], [6], [7], DNA repair and cell cycle [8], [9], insulin resistance, obesity and glucose levels [10], and inflammation [11], [12]. However, the possible association between taste receptors, bitter sensing and CRC risk has been recently tested [13], [14], [15], [16]. The gustatory system, developed during evolution, is a nutritional gatekeeper of the body to determine which food should be ingested and which should be rejected as potentially harmful. In particular, the capability to discriminate bitter taste has evolved as a central warning signal against the ingestion of possible toxic substances. *TAS2R38,* the most studied gene belonging to this family, is greatly involved in the ability to taste the glucosinolates, a large family of bitter tasting compounds which are widely distributed in plants and particularly in the *Brassica* sp. [17], [18]. The discrimination of bitter taste can influence the consumption of vegetables containing these ligands and since a reduced vegetable intake can increase colon cancer risk, individual responsiveness to bitter compounds could modulate the disease risk [19], [20], [21], [22]. Moreover, bitter taste receptors encoded by TAS2R gene family are expressed not only in the tongue but also in the cells of the gastrointestinal (GI) tract [23], [24]. Taste receptors may provide, in this way, the organism with two lines of defence against possible toxic agents. The first may be at the level of taste in the tongue, by avoiding ingestion. The second line of defence, the molecular sensing in the GI tract, could be responsible for the detection of ingested harmful drugs and toxins, thereby initiating responses critical for survival. Namely, the detection process is initiated by sending messages to the nervous system to give rise to the appropriate response of their neutralization and expulsion [23]. Furthermore, the taste sensing is an important system to start hormonal and/or neural pathways leading to the regulation of caloric intake, pancreatic insulin secretion, and metabolism [23]. Although these fundamental control systems have been known since many years, the cellular and neural pathways that mediate biological responses to luminal stimuli in general, and bitter stimuli in particular, remain poorly characterized, However, recent reports are quickly adding new information to this very important topic [25], [26], [27], [28].

*TAS2R38* gene is characterized by three non synonymous coding SNPs (rs713598 – G145C, Ala49Pro; rs1726866 – T785C, Val262Ala; rs10246939 – A886G, Ile296Val). These three polymorphisms, which are also tagging SNPs and cover all the common genetic variability of the gene locus, give rise to several haplotypes. Two of these haplotypes, Pro-Ala-Val (PAV) and Ala-Val-Ile (AVI) are by far the most commonly found in human populations [29], [30], [31]. Subjects possessing at least one copy of the PAV allele are significantly more responsive to bitter tastants, like PROP or PTC (taster phenotype), than those who are homozygous for the AVI allele (non-taster phenotype). Since the distinct phenotypes (taster *vs* non-taster) reflect a differential receptor functionality, the inability to taste bitter compounds could be also a marker for an impaired function of the receptors in GI: non-taster individuals could react slower in eliminating xenobiotics in the gut and consequently being at higher risk for CRC.

The aim of this study was, using a tagging approach, to evaluate the possible correlation between all the common genetic variability of *TAS2R38* and the resulting “tasting ability” and CRC risk in a total of 1203 cases of colorectal cancer and 1332 controls from the German and Czech populations, which are known to exhibit one of the higher incidence of CRC [32].

## Results

In this case-control study we evaluated the variability in the *TAS2R38* gene in a group of subjects of German and Czech Caucasian origin. Details regarding the main characteristics of the two study populations are presented in Table 1.

**Table 1:** Characteristics of colorectal cancer patients and control subjects.

Populations		Czech		German		Czech+German	
		Cases	Controls	Cases	Controls	Cases	Controls
		(*n* = 601)	(*n* = 623)	(*n* = 602)	(*n* = 709)	(*n* = 1203)	(*n* = 1332)
Males/Females (%)*		57.3/42.7	53.9/46.1	49.7/50.3	49.2/50.8	53.5/46.5	51.4/48.6
**Age**	**Average**	59.2 yrs	55.3 yrs	43.2 yrs	44.5 yrs	51.2 yrs	49.6 yrs
	**Lower Quartile**	54.0 yrs	47.0 yrs	36.0 yrs	35.0 yrs	41.0 yrs	39.0 yrs
	**Upper quartile**	67.0 yrs	65.0 yrs	49.0 yrs	53.0 yrs	63.0 yrs	59.0 yrs

### Genotyping success rates and quality control

The genotype distributions at all loci were in Hardy-Weinberg equilibrium in controls, with non-significant chi square values (p>0.05, data not shown). Random duplicate samples (8%) were also included and concordance of their genotypes was greater than 99%. The average call rate for the three SNPs was 97.9%.

### Main effects of genotyped SNPs

The distribution of the genotypes and their odds ratios (ORs) for association with CRC risk are shown in Table 2. We evaluated the ORs separately and jointly for the two populations. We found that there were no significant associations between the SNPs of the *TAS2R38* gene and CRC neither in the Czechs, in the Germans, nor in the joint analysis.

**Table 2:** Associations of *TAS2R38* tagging polymorphisms with colorectal cancer risk.

	Czech population					German population					Combined population				
SNP	Cases^a^	Controls^a^	OR (95% CI)^b^	P_value_	P_trend_	Cases^a^	Controls^a^	OR (95% CI)^b^	P_value_	P_trend_	Cases^a^	Controls^a^	OR (95% CI)^b^	P_value_	P_trend_
**rs713598**	586	576			0.31	552	679			0.03	1138	1255			0.05
**C/C**	123	115	1			76	118	1			199	233	1		
**C/G**	272	303	0.87 (0.64–1.19)	0.47		259	327	1.23 (0.88–1.72)	0.22		531	630	0.98 (0.79–1.23)	0.90	
**G/G**	191	158	1.13 (0.80–1.58)	0.47		217	234	1.40 (0.98–1.98)	0.05		408	392	1.21 (0.96–1.54)	0.09	
**C/G+G/G**	463	461	0.96 (0.71–1.29)	0.80		476	561	1.30 (0.94–1.79)	0.10		939	1022	1.10 (0.89–1.36)	0.36	
**rs1726866**	584	572			0.40	555	677			0.07	1139	1249			0.05
**C/C**	105	97	1			104	155	1			209	252	1		
**C/T**	261	287	0.84 (0.61–1.18)	0.33		264	315	1.23 (0.91–1.67)	0.16		525	602	1.03 (0.83–1.29)	0.73	
**T/T**	218	188	1.05 (0.74–1.48)	0.76		187	207	1.30 (0.94–1.80)	0.10		405	395	1.20 (0.95–1.52)	0.11	
**C/T+T/T**	479	475	0.93 (0.68–1.26)	0.66		451	522	1.26 (0.95–1.67)	0.10		932	997	1.26 (0.89–1.35)	0.35	
**rs10246939**	584	590			0.27	531	686			0.06	1115	1276			0.04
**C/C**	121	118	1			102	155	1			223	273			
**C/T**	266	304	0.89 (0.65–1.22)	0.50		245	323	1.13 (0.83–1.54)	0.41		511	627	1.00 (0.80–1.24)	0.96	
**T/T**	197	168	1.14 (0.82–1.60)	0.41		184	208	1.29 (0.93–1.79)	0.11		381	376	1.23 (0.98–1.56)	0.07	
**C/T+T/T**	463	472	0.98 (0.73–1.32)	0.94		429	531	1.20 (0.90–1.59)	0.21		892	1003	1.09 (0.89–1.33)	0.39	

a Numbers may not add up to 100% of subjects due to genotyping failure. All samples that did not give a reliable result in the first round of genotyping were resubmitted to up to two additional rounds of genotyping. Data points that were still not filled after this procedure were left blank.

b OR: odds ratio; CI: confidence interval.

### Haplotype, diplotype and phenotype analysis

The distribution of the major haplotypes (PAV and AVI) was not significantly different (p = 0.99) in the two populations. Although rare haplotypes (AAV; PVV; AAI; PVI and PAI) accounted for the same cumulative frequency (4%) for both the German and the Czech groups, they appeared differently distributed as reported in supplementary Figures S1 and S2. Performing logistic regression analysis jointly on the German and the Czech populations we observed a statistically significant association between *TAS2R38* diplotypes and CRC risk: carriers of the AVI/AVI diplotype present an increased risk of CRC compared to the PAV/PAV carriers with an OR of 1.33 (95% CI 1.03–1.72; p = 0.027). Country of origin and gender did not significantly modify the observed association (p = 0.85 and p = 0.36 respectively) as reported in Table 3.

**Table 3:** Associations of the *TAS2R38 common* diplotypes with colorectal cancer risk.

	Czech population					German population					Combined population				
Diplotype	Cases^a^	Controls	OR (95%)^b^	P value	P trend	Cases^a^	Controls	OR (95%)^b^	P value	P trend	Cases^a^	Controls	OR (95%)^b^	P value	P trend
**PAV/PAV**	93	95	1		0.187	62	118	1		0.009	155	213	1		0.007
**PAV/AVI**	210	259	0.84 (0.60–1.19)	0.341		190	293	1.22 (0.85–1.75)	0.265		400	552	1.00 (0.78–1.28)	0.948	
**AVI/AVI**	176	155	1.15 (0.80–1.66)	0.436		161	198	1.52 (1.05–2.21)	0.026		337	353	1.33 (1.03–1.72)	0.027	
**PAV/AVI+AVI/AVI**	386	414	0.96 (0.69–1.32)	0.819		351	491	1.34(0.96–1.88)	0.084		737	905	1.13(0.90–1.42)	0.278	

a Numbers may not add up to 100% of subjects due to genotyping failure. All samples that did not give a reliable result in the first round of genotyping were resubmitted to up to two additional rounds of genotyping. Data points that were still not filled after this procedure were left blank.

b OR: odds ratio; CI: confidence interval.

In the Czech population the carriers of the AVI/AVI diplotype presented a statistically not significant tendency to increased CRC risk, with an OR of 1.15 (95% CI 0.80–1.66; p = 0.44).

In the German population we found a significant association between the AVI/AVI diplotype carriers and increased risk of CRC with an OR of 1.52 (95% CI 1.05–2.21; p = 0.027) compared to the PAV/PAV carriers.

Finally, we divided all the diplotypes in two groups defined by their phenotype, the taster (PAV/PAV; PAV/PVV; P_*/A_*)) group and the non-taster ((AVI/AVI; AAV/AVI) group. Non-tasters were associated with an increased risk of CRC in the German population (OR = 1.36; 95% CI 1.06–1.75; p = 0.016). Non-tasters were also associated with an increased risk in the Czech population, although not at a statistically significant level (OR = 1.28; 95% CI 0.99–1.67; p = 0.0580. Analyzing the two populations together the association was stronger (OR = 1.34; 95% CI 1.12–1.61, p = 0.001.(Table 4)

**Table 4:** Associations of bitter sensing phenotypes with colorectal cancer risk.

	Czech population(n = 1069)				German population(n = 1091)				Czech+German population(n = 2160)			
TASTER	Cases	Controls	OR (95%)	P value	Cases	Controls	OR (95%)	P value	Cases	Controls	OR (95%)	P value
PAV/PAV+PAV/PVV+P_*/A_*	343	395	1		260	436	1		603	831	1	-
**Non-TASTER**												
AVI/AVI+AAV/AVI	176	155	1.28 (0.99–1.67)	0.058	178	217	1.36 (1.06–1.75)	0.016	354	372	1.34 (1.12–1.61)	0.001

a Numbers may not add up to 100% of subjects due to genotyping failure. All samples that did not give a reliable result in the first round of genotyping were resubmitted to up to two additional rounds of genotyping. Data points that were still not filled after this procedure were left blank.

b OR: odds ratio; CI: confidence interval.

### Effects of genotyped SNPs in different population strata

For the Czech population we have performed analysis stratifying for gender and smoking status and we also performed an analysis using BMI as adjustment factor. For the German population we did not have data on smoking and BMI. In our study there was no difference in the genotype distributions in the various strata, nor BMI had any effect on the association.

## Discussion

In the present study common genetic variation in *TAS2R38* was completely captured. The applied intensive SNP tagging approach provides a close to exhaustive analysis of associations of CRC risk with common polymorphic variants known for the locus of interest. Moreover the analyses of haplotypes, diplotype and phenotype provide a comprehensive picture of the common genetic variability of the *TAS2R38* gene in relation with CRC risk.

In this study we had sufficient power (over 80% for a codominant model) to detect OR = 1.30 at alpha = 0.05 for a SNP with a MAF of 0.33 (which is the most rare allele hereby studied) if considering only the German population. For the Czech population we had the same power for detecting associations of OR = 1.27 or greater and pooling together the two populations we obtained sufficient power to detect more modest associations (OR≥1.21).

The present study provides interesting evidence on the possible role of TAS2R38 in CRC risk. Analyzing the three SNPs separately we did not find any association with CRC risk. However, when we analyzed the distribution of major diplotypes between the cases and the controls we found that the AVI/AVI combination is associated with an increased CRC risk. This association was stronger when considering the two population together (p for the trend test = 0.007). Individuals carrying this diplotype are referred as bitter compounds “non-tasters”, as opposed to “taster”. We finally considered the non-taster phenotype against all the others and found that it was associated with increased CRC risk in both populations, although the association was stronger in the German population than in the Czech. This association suggests that the distinct phenotypes, which reflect a differential receptor functionality in the inability to taste bitter compounds, could be also a marker for an impaired function of the receptors in GI: non-taster individuals could react slower in eliminating xenobiotics in the gut and consequently being at higher risk of CRC.

Possible limitations of this study include the relatively small sample size and the possible differences between the two selected populations, especially in the regard of possible confounding factors such as the diet. However, we can confidently exclude a major role of ethnic differences between the two populations. In a recent study Nelis and colleagues investigated the underlying population stratification in Europe showing that there were very little, if any, differences in the genetic make-up of Germans and Czechs [32]. Moreover, the Czech and the German populations also have a comparable CRC incidence according to Globocan [1]. Dietary habits and food intake are not dramatically different in the two countries (http://faostat.fao.org/site/609/DesktopDefault.aspx?PageID = 609). However we found a weaker association between the genetic variability of the *locus* and CRC risk in the Czech population. One explanation may be related to environmental factors (e.g. diet, smoking). A second explanation could be the different enrollment strategy of study subjects since Germans had a family history of CRC or CRC diagnosed under the age of 50. Mean and median age are lower in the German population and this may reflect the fact the causality of the tumour might have a stronger genetic component since the environment had less time to modify the cancer risk for these individuals. It is also possible that the interactions between genes and environment could be the cause of the weaker association in the Czech population in a way that we could not detect within this study. Finally the association could be a chance finding, but we would tend to exclude this for the fact that in the two populations the association seems to indicate the same group as the increased risk individuals.

Gender differences for bitter tastes have been explored [33] and for these reason we conducted stratified analyses in both populations. We found that sex was not a modifying factor in either population.

Several genome-wide association studies (GWAS) on CRC risk have been published [34], and in none *TAS2R38* emerged as a possible susceptibility locus. However, it is interesting to note that the genomic region in which the *TAS2R38* lies is poorly covered by the SNP arrays used in published CRC GWAS. In particular, rs1726866 and rs10246939 are not present on those platforms (http://genome.ucsc.edu/cgi-bin/hgGateway). We found an association by using the combinations of the three genotypes into diplotypes, therefore it is not surprising that GWAS, where two of the three SNPs were lacking, and which do not routinely study haplotypes/diplotypes/phenotypes, did not detect any signal at this locus.

In conclusion, we found a suggestive association between the human bitter tasting phenotype and the risk of CRC in two different populations of Caucasian origin. A larger, independent study is needed to further investigate this finding.

## Materials and Methods

### Ethics Statement

All participants signed an informed written consent. The study was approved by the ethical review boards of the institutions responsible for subject recruitment in each of the recruitment centres.

Written informed consent was obtained from all study participants. The ethical committees were the following:

Ethik Kommission der Medizinischen Fakultät der Ruhr Universität Bochum [Reg.-Nr.:1514]; Ethik Kommission – Medizinische Fakultät Bonn [Lfd. Nr. 115/09]; Ethik Kommission der Medizinische Fakultät der Technischen Universität; Dresden [Bearbeitungs- Nr. EK170102000]; Ethikkommission der Medizinische Fakultät der Heinrich Heine Universität Düsseldorf [Studiennummer: 1172]; Ethikkommission I der Universität - Medizinische Fakultät Heidelberg [Antrags- Nr.: 220/2002]; Ethikkommission II an der Fakultät für Klinische Medizin der Ruprechts-Karl-Universität Heidelberg (concerning samples from Mannheim) [Antrags- Nr.: 87/04] Ethikkommission der Medizinische Fakultät Universität München [Projekt Nr. 255/98]; Etická komise Ústravu experimentální medicí AV ČR Ethics Committee of the Institute for clinical and Experimental Medicine and Faculty Thomayer Hospital [Č.j. 786/09 (09-04-09)].

### Study populations

For the present cases-control study we have considered a group of subjects from two populations: one from Czech Republic (601 cases, 623 controls) and the other from Germany (602 cases, 709 controls).

The Czech population has been extensively described elsewhere [7]. Briefly, cases were CRC patients visiting nine oncological departments (two in Prague, one each in Benesov, Brno, Liberec, Ples, Pribram, Usti nad Labem, and Zlin) distributed in all geographic regions of Czech Republic and being representative of the population of the entire country. This study includes patients who could be interviewed and provided biological samples of sufficient quality for genetic analysis. All cases had histological confirmation of their tumor diagnosis. In the group of cases, genetic testing for hereditary nonpolyposis CRC (HNPCC) was recommended to four patients, who belonged to families complying with the Amsterdam criteria II, and these cases were excluded.

Controls were selected among patients admitted to five large gastroenterological departments (Prague, Brno, Jihlava, Liberec, and Pribram) all over the Czech Republic, during the same period as the recruitment of cases. Only subjects whose colonoscopic results were negative for malignancy, colorectal adenomas or IBD were chosen as controls. Among 739 invited controls, a total of 623 (84.3%) were analyzed in this study (lost controls were similar to those included with respect to sex distribution).

Cases included in this study had a mean age of 59.2 years (range 27–74), while controls had a mean age of 55.3 years (range 28–91).

The genetic analyses did not interfere with diagnostic or therapeutic procedures for the subjects. All participants signed an informed written consent and the design of the study was approved by the Ethical Committee of the Institute of Experimental Medicine, Prague, Czech Republic.

For the German population, as described in [35], [36] CRC cases comprised 602 index patients (age range 13–82 years, mean 43.2 years) recruited by six German university hospitals (Bochum, Bonn, Dresden, Düsseldorf, Heidelberg and Munich/Regensburg). Cases were collected as part of a large study on susceptibility to HNPCC. Inclusion criteria for the cases were (i) a family history of CRC or (ii) CRC diagnosed under the age of 50. Analysis for microsatellite instability was applied as a pre-screening test prior to mutation analysis in the *MSH2* and *MLH1* genes. All cases were tested to be microsatellite stable.

The control series consisted of 709 healthy, unrelated and ethnicity-, sex- and age-matched blood donors (26–64 years, mean 44.5 years) which were recruited between 2004 and 2006 by the Institute of Transfusion Medicine and Immunology, Faculty of Mannheim, Germany. The matching intervals for age were ‘younger than 30 years’, five-year groups (30–34, 35–39,…,60–64) and ‘older than 65 years’. Blood sampling was performed during regular blood donation according to German guidelines. The study was approved by the competent local Ethics Committees, and written informed consent was obtained from all individuals.

Details regarding the main characteristics of the two study populations are presented in Table 1.

### Selection of tagging SNPs

We aimed at surveying the entire set of common genetic variants in *TAS2R38*. To this end, we followed a hybrid tagging-functional approach. We used the algorithm described by Carlson and coworkers [37] that was developed to select the maximally informative set of tag SNPs in a candidate-gene association study. All polymorphisms in the region of *TAS2R38* locus with minor allele frequency (MAF) ≥5% in Caucasians from the International HapMap Project (version 21a; http://www.hapmap.org) were included. Tagging SNPs were selected with the use of the Tagger program within Haploview (http://www.broad.mit.edu/mpg/haploview/; http://www.broad.mit.edu/mpg/tagger/) [38], [39], using pairwise tagging with a minimum r^2^ of 0.8. Considering that the genomic region of *TAS2R38* is characterized by high levels of linkage disquilibrium (LD), we postulate that such SNPs are also likely to tag any hitherto unidentified common SNPs in the gene. We selected rs713598, rs1726866 and rs10246939 as tagging SNPs since they are all non-synonymous functional [29], [30], [40], [41], [42], [43], [44], [45] SNPs.

### DNA extraction and genotyping

DNA was extracted from blood samples with standard proteinase K digestion followed by phenol/chloroform extraction and ethanol precipitation. The order of DNAs of cases and controls was randomized on PCR plates in order to ensure that an equal number of cases and controls could be analyzed simultaneously. All genotyping was carried out using the Taqman assay. The MGB Taqman probes and primers were purchased from Applied Biosystems (Foster City, CA) as pre-designed assays. The reaction mix included 10 ng genomic DNA, 10 pmol each primer, 2 pmol each probe and 2.5 ml of 2x master mix (Applied Biosystems) in a final volume of 5 µl. The thermocycling included 40 cycles with 30 s at 95°C followed by 60 s at 60°C. PCR plates were read on an ABI PRISM 7900HT instrument (Applied Biosystems).

All samples that did not give a reliable result in the first round of genotyping were resubmitted to up to two additional rounds of genotyping. Data points that were still not filled after this procedure were left blank.

### Haplotype and diplotype reconstruction

Haplotypes and diplotypes were reconstructed using PHASE software [46].

### Statistical Analysis

The frequency distribution of genotypes was examined for the cases and the controls. Hardy-Weinberg equilibrium was tested in the cases and in the controls and in the two populations separately by chi square test. We used logistic regression for multivariate analyses to assess the main effects of the genetic polymorphism on CRC risk using a co-dominant and a dominant inheritance model. The most common genotype in the controls was assigned as the reference category. All analyses were adjusted for age and gender. In the Czech population we also adjusted for Body Max Index (BMI) as a continuous variable. We analyzed the two populations separately and together (we adjusted also for center of recruitment in the latter case).

Logistic regression considering the reconstructed haplotype adjusted for age (continuous), sex and study center was performed to calculate risk estimates. The “taster” (PAV) haplotype was set as reference group. We finally created diplotypes for each individual and grouped and divided them into two phenotypic groups, the taster group ((PAV/PAV; PAV/PVV; P_*/A_*) and the non-taster group (AVI/AVI;AAV/AVI). For the diplotype analysis the “taster” group was set as reference.

We also performed stratified analysis for gender and smoking habits. Smokers were classified as current smokers, ex smokers (quit smoking for more than 5 years) or never smokers. Smokers were subsequently divided in heavy smokers (more than 20 cigarettes per day) and non-heavy smokers (less than 20 cigarettes per day). All analysis were performed using STATGRAPHICS*®* Centurion XVI software (© 2009 by StatPoint Technologies, Inc.www.STATGRAPHICS.com) and STATA software (StataCorp, College Station, TX).

## Supporting Information

Figure S1 **Distribution of the Haplotypes in the Czech population.**
(DOC)

Figure S2 **Distribution of the Haplotypes in the German population.**
(DOC)
