# Supplementary figures and images for: Association Between TAS2R38 Gene Polymorphisms and Colorectal Cancer Risk: A Case-Control Study in Two Independent Populations of Caucasian Origin
Source: PLoS One. 2011 Jun 2;6(6):e20464. doi: 10.1371/journal.pone.0020464 (PMC3107225; doi:10.1371/journal.pone.0020464)

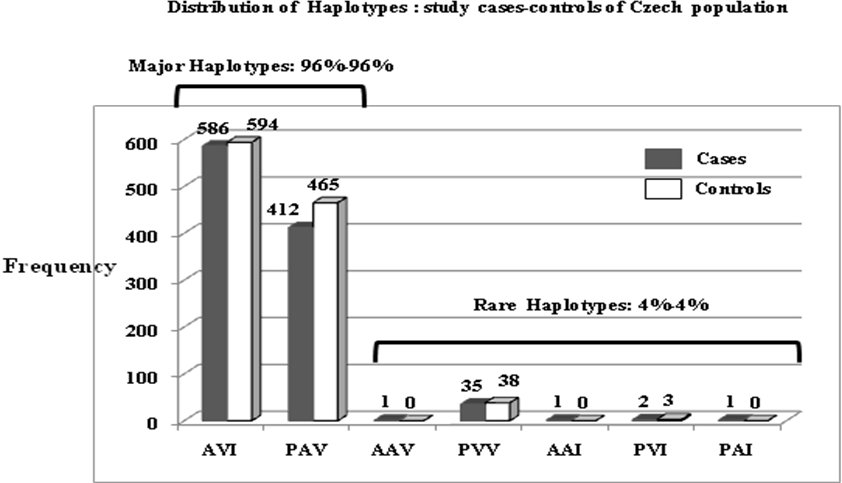

Supplement: Figure S1 — Distribution of the Haplotypes in the Czech population. (DOC) [file pone.0020464.s001.doc]

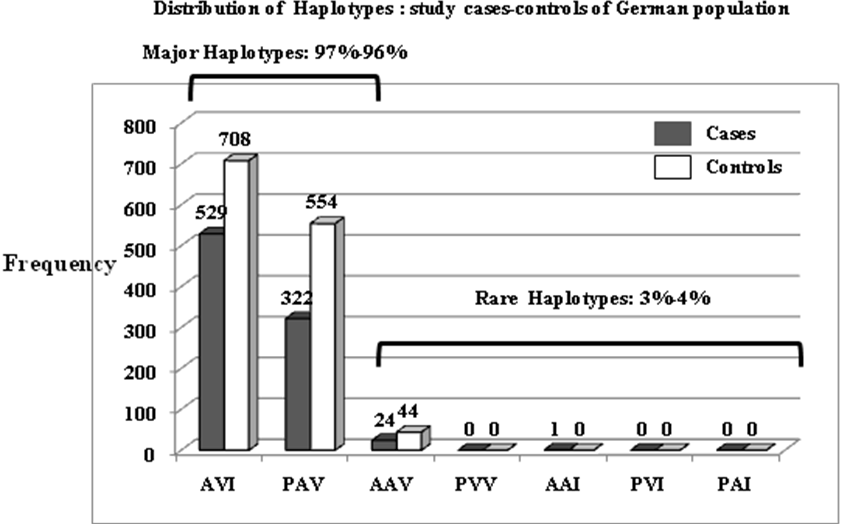

Supplement: Figure S2 — Distribution of the Haplotypes in the German population. (DOC) [file pone.0020464.s002.doc]
